# Anesthesia Assistants (Nurses and Non-nurses) in Japan: A Narrative Review

**DOI:** 10.7759/cureus.91321

**Published:** 2025-08-31

**Authors:** Susumu Yoshida, Kiyoyuki Miyasaka

**Affiliations:** 1 Perianesthesia Nursing, International University of Health and Welfare Graduate School, Tokyo, JPN; 2 Anesthesiology, St. Luke’s International Hospital, Tokyo, JPN

**Keywords:** anesthesia assistant, anesthesia care provider, anesthetist, perianesthesia medicine, task sifting

## Abstract

There is a marked shortage of anesthesiologists in Japan due to the aging population, with an increasing demand for anesthesia services. To shift the burden of intraoperative tasks from anesthesiologists, an increasing number of medical personnel, both nurses and non-nurses, have taken on responsibilities in anesthesia care. We conducted this narrative review to summarize the roles of professions involved in anesthesia assistance in Japan and to consider the standardization of surgical anesthesia delivery and personnel training, referencing global trends. A literature search was conducted using PubMed, the Japanese Igaku Chuo Zasshi database, and the websites of relevant professional associations using keywords relevant to assistance by non-physician providers during anesthesia care. Nurses are the major providers of anesthesia assistance. For nurses, there is a national training program that includes anesthesia-related tasks (Specified Medical Acts Training) as well as several other programs (Perianesthesia Nurse: PAN, Nurse Practitioner: NP, Certified Nurse: CN, Perioperative Management Team: PMT) accredited by various academic organizations. The scope of intraoperative anesthesia assistance is almost the same regardless of certification. Only PAN offers a postgraduate course in anesthesia nursing. For clinical engineers, PMT certification is available in addition to in-house facility certifications. Their anesthesia assistance duties are nearly the same as those provided by nurses. Many providers responsible for intraoperative anesthesia assistance are nurses, and their educational backgrounds or certification programs vary. It is expected that the national government and related organizations will integrate and standardize the qualifications and education of both nurses and clinical engineers.

## Introduction and background

In Japan, the number of surgical procedures is on the rise, along with an aging society and increasing workload on anesthesiologists involved in acute and intensive care and pain medicine. This has resulted in a serious shortage of anesthesiologists [1,2]. In 2007, the Japanese Society of Anesthesiology (JSA) began to advocate for the establishment of “perioperative management teams” led by anesthesiologists to ensure patient safety and quality of anesthesia, starting a certification system for medical personnel in 2014 [3]. In Japan, only physicians are permitted to administer anesthesia. Other medical personnel can provide assistance by shifting/sharing the anesthesiologist's tasks within their legal scope of practice. While the task of intraoperative patient monitoring has historically been shared with other providers, there was no systematic anesthesia education for non-anesthesiologist providers until recently [4]. A postgraduate education program for anesthesia care was launched for the first time in Japan in 2010 [4,5]. Currently, professionals with varying educational backgrounds and levels of certification may provide anesthesia assistance in the operating room [6]. We hope that this review of intraoperative anesthesia assistants will lead to further consideration of the future provision of anesthesia, the framework for education, and the development of human resources in Japan and other countries.

## Review

Methods

A narrative review was conducted in the absence of systematic literature on this topic. A literature search for articles concerning medical personnel involved in anesthesia assistance in Japan was conducted using PubMed and the Japanese Igaku Chuo Zasshi database in December 2024. The PubMed search was for English-language articles, using the following search keywords: anesthesia, anesthesia assistant, anesthesia care provider, anesthesiologist assistant, non-physician, nurse, anesthesia nurse, clinical engineer, pharmacist, Japan. The Japanese Igaku Chuo Zasshi database was searched using corresponding Japanese keywords: Masui (anesthesia), Masuihojo (anesthesia assistant), Kangoshi (nurse), Tasukusifuto (task shifting), Rinsyokogakugisi (clinical engineer), Yakuzaishi (pharmacist).

Following screening by the authors, 3 articles were identified using the PubMed search, and 21 articles from the Japanese Igaku Chuo Zasshi. We also searched the websites of relevant professional associations using keywords relevant to assistance by non-physician providers during anesthesia care, referencing a total of 24 sources.

Non-physician Providers of Anesthesia Care in Japan

Other than physicians, nurses, and clinical engineers are the only occupations that assist the anesthesiologist intraoperatively in Japan. There are no reports of pharmacists providing direct anesthesia assistance, such as patient monitoring, in the operating room due to their scope of practice.

1. Nurses

In Japan, registered nurses who have been trained in additional anesthesia-related educational programs include nurses who have completed training for “specified medical acts” as defined by the Ministry of Health, Labour and Welfare (MHLW), perianesthesia nurses, nurse practitioners, certified nurses, and perioperative management team nurses (Table 1).

**Table 1:** Nurses providing anesthesia assistance in Japan JSPANM: Japanese Society for Perianesthesia Nursing and Medicine ○ means “includes.” △ means “includes in part, but not sufficiently.”

	Nurses Trained in Specified Medical Acts	Perianesthesia Nurse (PAN)	Nurse Practitioner(NP)	Certified Nurse(CN)	Perioperative management team nurses
Year established	2015	2010	2008	2005	2014
Establishment organization	Ministry of Health, Labour, and Welfare	Japanese Society for Perianesthesia Nursing and Medicine	Japanese Organization Nurse Practitioner Faculty	Japanese Nursing Association	Japanese Society of Anesthesiology
Training Institution	289 designated training institutions (intraoperative anesthesia management package: 140 institutions) [7]	Graduate School of Nursing Master's Program: 6 schools (Yokohama City University, Shinshu University, Nara Medical University, International University of Health and Welfare, Nagoya City University, and Shiga University of Medical Science); Unpublished date from JSPANM [8]	Graduate School of Nursing Master's Program: 19 schools [9]	Japanese Nursing Association: 1 institution [10]	Japanese Society of Anesthesiology/Japan Perioperative Nursing Academy [3]
Training period	Varies depending on the number of specified medical acts and training institutions [11]	2 years (master’s degree) [8]	2 years (master’s degree) [9]	1 year [10]	Not applicable
Educational program on anesthesia knowledge and skills	None [11]	○ [8]	△ [9]	△ [10]	△ [3]
Specified medical acts	○ [11]	○ [8]	○ [9]	○ [10]	None
Number of completed courses	8820, 852: Anesthesia package (Aug 2023）[7]	72 (April 2025), Unpublished data from JSPANM [8]	984 (April 2025) [12]	787 (Dec 2024) [10]	2063 (April 2025) [13]

Nurses who completed training pertaining to specified medical acts:* *The training system for nurses related to specified medical acts, implemented by the MHLW in 2015, allows trained nurses to perform several specified medical acts (38 acts in 21 categories, Table 2), within the confines of established procedure manuals, which serve as standing orders given by physicians in advance.

**Table 2:** Specified Medical Acts (38 acts in 21 categories), translation by MHLW This entire table is quoted from reference [11]. ＊Eight acts included in the intraoperative anesthesia management package MHLW: Ministry of Health, Labour, and Welfare

Category	Medical Acts
Respiratory (acts pertaining to airway management)	Positioning of oral/nasal tracheal intubation tubes*
Respiratory (acts pertaining to artificial respiratory therapy)	Changing invasive positive pressure ventilation settings*
	Changing non-invasive positive pressure ventilation (NPPV) settings
	Adjusting sedation dosage for patients on mechanical ventilation
	Weaning patients from mechanical ventilation*
Respiratory (acts pertaining to long-term respiratory therapy)	Replacement of tracheal cannulae
Artery blood gas analysis	Blood sampling by direct arterial puncture*
	Placement of radial arterial catheters*
Circulatory	Operation and management of temporary pacemakers
	Removal of temporary pacemaker wires
	Operation and management of assisted circulation devices (e.g., percutaneous cardiopulmonary support (PCPS))
	Adjustment of assistance frequency for weaning from intra-aortic balloon pumping (IABP)
Dialysis management	Operation and management of dialysis/diafiltration devices pertaining to acute blood purification
Intraperitoneal drain management	Removal of intraperitoneal drains (including removal of intraperitoneal puncture needles)
Chest tube management	Removal of chest tubes
	Setting and adjustment of continuous low-pressure suction devices
Pericardial drain management	Removal of pericardial drains
Postoperative pain management	Administration and adjustment of analgesics via epidural catheters*
Wound drain management	Removal of drains
Wound management	Removal of avascular necrotic tissue for treatment of bedsores or chronic wounds
	Negative pressure wound therapy
Drug administration pertaining to hemodynamics	Adjustment of continuous infusion of antihypertensive drugs
	Adjustment of the continuous infusion of catecholamines
	Adjustment of the continuous infusion of antihypotensive drugs
	Adjustment of the continuous infusion of diuretics
	Adjustment of the continuous infusion of electrolytes (K, Cl, Na)
	Adjustment of the continuous infusion of fluids with glucose and/or electrolytes*
Drug administration pertaining to glycemic control	Adjustment of insulin dosage
Drug administration pertaining to nutritional and/or fluid management	Assessment of the degree of dehydration and correction by fluid administration*
	Adjustment of continuous infusion of total parenteral nutrition
Catheter management pertaining to nutrition (central venous catheters)	Removal of central venous catheters
Catheter management pertaining to nutrition (PICC)	Insertion of PICC (peripherally inserted central catheters)
Drug administration pertaining to psychiatric and neurologic symptoms	Pro re nata administration of anticonvulsant drugs
	Pro re nata administration of antipsychotic drugs
	Pro re nata administration of anxiolytic drugs
Drug administration pertaining to infection	Pro re nata administration of drugs for patients with signs of infection
Drug administration pertaining to skin injury	Subcutaneous steroid dosing and injection for extravascular infiltration of chemotherapy and other drugs
Fistula management	Replacement of gastric/enteric fistula tubes or gastrostomy buttons
	Replacement of vesical fistula catheters

Under this legislation, some advanced medical acts that require direct physician supervision may be performed by nurses [11]. The original intent of this system was to grant greater autonomy to nurses in the home and community healthcare setting. From 2019, training packages were announced for other clinical settings that bundle training for several specified acts likely to be required in those settings. One of these is the “intraoperative anesthesia management” package, which includes training for eight acts in six categories: re-positioning intubation tubes, changing ventilator settings and weaning, arterial sampling and line placement, administering fluid with glucose and/or electrolytes, and administering analgesics through epidural catheters. Despite the package name, training does not include any content on anesthesia management, such as pharmacology and pathophysiology, respiratory and circulatory management, patient monitoring, and crisis management. As of September 2024, 140 institutions and organizations are offering specific medical acts training, with the intraoperative anesthesia management package being the most popular. A total of 287 trained nurses are active in operating rooms all over Japan as of 2022 [7], with an additional 852 nurses completing this package of training in 2023. However, the total number of nurses trained under this initiative is 8,820, far short of MHLW’s target of 100,000 by 2025.

Perianesthesia nurse (PAN): The PAN is an advanced practice nurse who provides anesthesia care throughout the perioperative setting [4]. In Japan, this is the only program that offers postgraduate education specifically for nurses specializing in anesthesia. The first PAN master’s degree program was established at St. Luke's International University in 2010, and is now offered at six graduate schools [4,14]. Another graduate school offers a similar perioperative education program. These programs are characterized by having anesthesiologists as faculty teaching basic medical sciences such as anatomy and physiology, pharmacology, and clinical reasoning. Training for specified medical acts (i.e., the intraoperative anesthesia management package) may also be included. PANs are dependent providers who only work in tandem with an attending anesthesiologist under their direct orders and supervision. PANs may be supervised to perform intubation and extubation (as part of their ongoing on-the-job training to assist anesthesiologists), change ventilator settings, and adjust doses of anesthesia-related medications. Their scope of clinical practice is not limited to intraoperative anesthesia but extends to activities throughout the perianesthesia period, including preoperative outpatient anesthesia, postoperative pain management, obstetric anesthesia services, sedation outside of the operating room, outpatient pain clinic, and so on. In September 2024, the Japanese Society for Perianesthesia Nursing and Medicine began a certification system [15], which includes a written examination following graduation from an approved degree-granting program.

Nurse practitioner (NP):* *NPs are nurses who can provide a certain level of care based on ethical and scientific evidence in cooperation and collaboration with physicians and other professionals to improve patient quality of life [9]. Training in Japan was first started in 2008 at the Oita Prefectural College of Nursing Graduate School Master's Course, and currently, 19 graduate courses are offered. In addition to basic nursing courses, the curriculum emphasizes physical assessment, pharmacology, and pathophysiology to enable graduates to manage patient symptoms autonomously and in a timely manner. Programs are offered in primary care or critical care and cover a variety of disease processes and treatments ranging from acute to home care, as well as training in the specified medical acts. To be certified as an NP, graduates of approved programs must pass a written examination administered by the Japanese Organization Nurse Practitioner Faculties. According to a 2017 survey on where NPs work, 80% were employed by hospitals, with the majority affiliated with medical departments such as emergency and critical care, surgery (respiratory, orthopedic, digestive), and internal medicine (respiratory, general, cardiovascular) rather than nursing. Some NPs are also involved in surgical anesthesia [16]. NPs typically provide anesthesia care under the direct supervision of anesthesiologists, and in this sense, their scope of practice is similar to that of PANs [17,18].

Certified nurse (CN) in perioperative nursing: The Japanese Nursing Association certifies nurses in 21 fields, including perioperative nursing. The CN is expected to provide a high standard of nursing care, as well as play a role in providing education and consultation for other nurses [15]. Certification in perioperative nursing began in 2005, and the curriculum was updated in 2020 to include training in the specified medical acts (i.e., the intraoperative anesthesia management package). The new one-year curriculum for perioperative nursing focuses mainly on the specified medical acts, and only includes one course on anesthesia. With an ongoing shortage of operating room nurses, CNs in perioperative nursing are typically unable to specialize in anesthesia-related duties [19]. Outside the operating room, CNs in perioperative nursing may be involved in the preoperative outpatient clinic and postoperative pain management [20].

Perioperative management team (PMT) nurse:* *The JSA has been advocating the “perioperative management team” concept since 2007 to improve the quality of perioperative care. Following the establishment of seminars, textbooks, and various educational activities, certification of nurses as PMT members began in 2014, with the addition of pharmacists in 2016, and clinical engineers in 2017 [3]. Certification is proof of education in basic perioperative concepts and a willingness to promote team-based medicine in the operating room. The roles expected of PMT nurses include double-checking anesthesiologists' work, evaluating perioperative risks, and coordinating with other medical staff. In some facilities, they provide support for intraoperative anesthesia under the direct supervision of anesthesiologists. Specified medical acts training is not included in PMT certification and must be taken separately. It is unclear how many PMT nurses are also trained in specified medical acts or have received additional on-the-job training relevant to anesthesia.

2) Clinical Engineer

Clinical engineer anesthesia assistants (CEAA): Institutional initiatives exist for clinical engineers who wish to specialize in anesthesia care. In 2010, Nara Medical University began training clinical engineers as anesthesia assistants [5]. Similar training programs exist in multiple facilities in Japan, typically consisting of lectures and on-the-job training for six months to one year (depending on the facility). Those who pass the in-house certification examination are certified by the facility. Examples of duties performed in the operating room include preparation of anesthesia equipment and airway devices, placing monitors on patients, assisting with insertion of peripheral intravenous lines, charting anesthesia records, changing ventilator settings, adjusting drug doses, monitoring vital signs, taking arterial blood samples, and assisting with intubation [21]. During the maintenance phase of anesthesia, the CEAA may be the only anesthesia provider in the room [22]. In 2021, the Japanese Clinical Engineer Act was revised, allowing them to secure peripheral venous lines and operate the anesthesia machine under the direction of a physician [23]. The use of CEAAs appears to be on the rise, but it is unclear exactly how many are currently certified.

Perioperative management team (PMT) clinical engineer: As previously described, the JSA began certifying clinical engineers as part of its “perioperative management team” initiative in 2017. Certification requires three years of clinical experience in the perioperative field, as well as attendance in seminars and e-learning courses sponsored by the society, and passing a written examination. As of April 2025, there are 269 clinical engineers who have received PMT certification [24]. The expected roles of PMT clinical engineers include checking anesthesia machines and monitoring equipment, responding to crises, preparing patient-controlled analgesia (PCA) equipment, and organizing medical information. The reality of PMT clinical engineers’ scope of practice remains unclear, including how much overlap there is with CEAAs.

Discussion

Summary of Key Findings

Japan faces a serious shortage of anesthesiologists and is looking to non-physician practitioners such as nurses and clinical engineers for assistance. Nurses with various certifications, including PAN, NP, and the National Specified Medical Acts Training program, provide anesthesia assistance, including intraoperative tasks. Among these, the PAN program is unique, as it offers formal postgraduate-level education in anesthesia. Clinical engineers also assist, certified through the PMT program or in-house programs. A key concern is the heterogeneity of training, prompting calls for standardization. While task-shifting alleviates the burden on anesthesiologists, there is a lack of high-quality studies on its impact on patient safety. Further research is needed to evaluate the outcomes of this practice.

Shortage of Anesthesiologists

MHLW has long maintained the position that there is no need to increase the number of physicians because Japan’s population is decreasing. MHLW projects that physician supply will exceed demand sometime around 2030 [25]. In the meantime, ongoing shortages of physicians and excessive physician work hours have prompted the MHLW to promote work style reform. In the field of anesthesia, this involves initiatives to shift the burden of time-intensive tasks such as intraoperative management from physician anesthesiologists to other medical providers [26]. Globally, the recommended number of anesthesiologists is 20 per 100,000 population [27]. Japan is still far short of this standard at 8 per 100,000 population, despite recent increases in the number of anesthesiologists [28]. Task-shifting to non-physician providers may help reduce overtime and may allow anesthesiologists to venture outside the operating room to provide anesthesia care, as well as education and research. The JSA maintains that only physician anesthesiologists should perform anesthesia, but is considering non-physician providers in discussions of task-shifting. A 2015 survey noted that while patients undergoing general, spinal, or epidural anesthesia are almost always managed by physician anesthesiologists, many facilities, even in urban centers, rely on outside help to do so [29]. The shortage of physician anesthesiologists remains an ongoing issue.

Heterogeneity of Training

Various training and certification programs exist for nurses. Some facilities allow operating room nurses without special postgraduate training to assist intraoperative anesthesia, but the actual state of current practice is unknown. Currently, the only legal basis for advanced medical practice by nurses in Japan is the specified medical act training system, but there are concerns that the prescribed training does not include sufficient knowledge and skills related to anesthesia [30]. Additional anesthesia content, as well as on-the-job training by physician anesthesiologists, may be appropriate. The reasons why certification and qualifications for nurses have not yet been standardized may be due to the legal framework of the Japanese health care system, which assumes that only physician anesthesiologists perform anesthesia. Efforts to involve other professionals to assist have historically been initiated by necessity at individual facilities, making it difficult to consolidate these local initiatives into a single nationwide qualification.

The JSA advocates for safe and high-quality anesthesia practice by physician anesthesiologists. In a statement on the role of nurses who have completed specified medical acts training, the JSA clearly states that nurses assigned to cases must be supervised 1:1 by a physician anesthesiologist, who should not be supervising multiple rooms simultaneously [31]. Task-shifting does not aim to delegate intraoperative anesthesia management but to serve as an additional layer of quality and safety. When nurses take on the task of intraoperative anesthesia monitoring, anesthesiologists gain a degree of physical freedom from the operating room. Simply being able to step out of the room reduces the burden on anesthesiologists and allows them to safely address other tasks, contributing to the quality of perianesthesia care as a whole. The largest contingent of nurses involved in anesthesia tasks in Japan are those who have completed specified medical acts training, compared to only a small number of PANs who have received anesthesia training at the postgraduate level. However, in addition to clinical duties, many PANs are active in education and research [6,14]. In the future, PANs are expected to play a role as leaders and coordinators among nurses who provide anesthesia support in Japan [6]. However, PANs are few in number and not readily differentiated from other qualified nurses. It may be helpful to survey career development after certification, such as appointment to leadership and management roles as advanced practice nurses.

Those on the receiving end of task-shifting also need to be considered. With a nationwide shortage of operating room nurses, there is concern that providing anesthesia assistance may lead to disruptions in operating room nursing coverage [32]. According to a 2024 report, 54% of facilities had nurses who had completed specified medical acts training providing anesthesia assistance in the operating room, though 45% of them did so less than one day a week [33]. How to recruit and train nurses to provide anesthesia assistance is a nationwide challenge. Efforts to unify certifications by various entities may also need to be considered by professional societies or at the national level.

In Japan, there have been no studies with a high level of evidence evaluating whether the quality and safety of anesthesia are improved when nurses are directly involved in anesthesia. Retrospective studies have reported reduced anesthesia induction times when NPs and anesthesiologists work together [17], and no difference in the frequency of intubation complications between PANs and late-career anesthesia residents [34]. Further studies evaluating the outcomes of task-shifting need to be conducted.

The shortage of physicians will continue to drive task-shifting to other medical personnel. However, many facilities are facing shortages of nurses and clinical engineers as well, making it difficult for them to take on new tasks. It is also necessary to consider the burden and anxiety of non-physician providers taking on advanced medical tasks typically performed by physicians. Anesthesia assistance is not only about the intraoperative setting, but also concerns the overall continuum of care of anesthesia. Effective use of human resources, including task-shifting, needs to be considered from a comprehensive viewpoint. Studies on the safety of shifting anesthesiologists' tasks are also needed. This outcome has not been clarified and needs to be included in the discussion of task shifting.

Anesthesia Care Providers in Other Countries

A recent international survey of anesthesia providers indicates that nurses directly administer anesthesia in some developing countries where there are no anesthesiologists or a shortage of anesthesiologists [35]. In developed nations, only Certified Registered Nurse Anesthetists (CRNAs) in the United States provide anesthesia independently. CRNAs are responsible for anesthesia under the supervision of an anesthesiologist or surgeon, but 30 states across the U.S. have legally relieved physicians of their duty to supervise CRNAs, accelerating their independence from physicians [36]. CRNA programs will completely transition from master's degree programs to doctoral programs in 2025 [37]. A Cochrane review compared whether there is a difference in mortality and complication rates between anesthesia managed by CRNAs alone and those managed under physician anesthesiologist supervision. The results are inconclusive due to the lack of high-quality studies [38]. CRNAs are unique to the U.S., and their practice model is not appropriate for Japan, where only physicians can legally administer anesthesia. Although the qualification system is different, the research methodology examined the impact on patient outcomes depending on whether the anesthesia procedures are performed by physicians or by CRNAs. For example, the development of CRNAs can be used as a reference for research that should be conducted by providers such as PANs in Japan.

Anesthesiologist assistants (AAs) in the U.S. are not nurses but provide anesthesia assistance under the supervision of physician anesthesiologists [39]. The AA completes a postgraduate master's degree program that is led by an anesthesiologist. A study comparing cases managed by CRNAs and anesthesiologists with those managed by AAs and anesthesiologists showed no difference in anesthesia outcomes such as mortality, hospital days, and medical costs [40]. In Europe, nurses with practice models similar to AAs are involved in anesthesia in some countries, such as France, Norway, and Sweden (and in Germany and Spain in a limited capacity) [41]. PANs in Japan also share a similar scope of practice with AAs. In developed countries, anesthesia education at the postgraduate level is common among providers expected to be directly involved in anesthesia assistance [41,42]. In considering the future direction of Japan, it will be useful to follow such trends in developed countries [43].

## Conclusions

A continuing shortage of anesthesiologists is expected to drive task-shifting to nurses and clinical engineers. Currently available training and certification systems for nurses and clinical engineers providing anesthesia assistance are diverse, and it is desirable to consider standardization of certification standards by the government and professional organizations. Research on the safety and effectiveness of anesthesia assistance by nurses and clinical engineers is lacking, and further evaluation is required in the future.
